# PPARs Mediate Lipid Signaling in Inflammation and Cancer

**DOI:** 10.1155/2008/134059

**Published:** 2008-12-21

**Authors:** Liliane Michalik, Walter Wahli

**Affiliations:** Center for Integrative Genomics, National Research Center Frontiers in Genetics, University of Lausanne, 1015 Lausanne, Switzerland

## Abstract

Lipid mediators can trigger physiological responses by activating nuclear hormone receptors, such as the peroxisome proliferator-activated receptors (PPARs). PPARs, in turn, control the expression of networks of genes encoding proteins involved in all aspects of lipid metabolism. In addition, PPARs are tumor growth modifiers, via the regulation of cancer cell apoptosis, proliferation, and differentiation, and through their action on the tumor cell environment, namely, angiogenesis, inflammation, and immune cell functions. Epidemiological studies have established that tumor progression may be exacerbated by chronic inflammation. Here, we describe the production of the lipids that act as activators of PPARs, and we review the roles of these receptors in inflammation and cancer. Finally, we consider emerging strategies for therapeutic intervention.

## 1. INTRODUCTION

Signal lipids are known to trigger systemic physiological responses, to control inflammatory reactions, and to regulate key cellular processes, such as cellular energy metabolism, cell survival, proliferation, migration, and differentiation [1]. Among these lipids, fatty acids, diverse fatty acid derivatives, some eicosanoids, and sterol derivatives are modulators of gene expression via binding and activation of the nuclear hormone receptors (NHRs) peroxisome proliferator-activated receptors (PPARs), liver X receptors (LXRs), and farnesoid X receptor (FXR) [2]. These transcription factors control genes that regulate lipid homeostasis [2] and, for PPARs in particular, inflammatory responses [3]. Disturbance of lipid signaling and/or NHR pathways promotes the progression of a long list of imbalances and diseases, such as obesity, type 2 diabetes, chronic inflammation, cardiovascular diseases, cancer, hypertension, degenerative diseases, autoimmune diseases, and a few others [1, 2]. Important cross-regulation exists between lipid signaling and NHR pathways, which generates a variety of responses dependent on signaling networks that are often tissue-specific [1].

In this paper, we propose an integrated view of the production of the lipids that activate PPARs, and of the functions of these receptors in inflammation and cancer. We conclude with comments on therapeutic opportunities.

The three PPAR isotypes (PPAR*α* or NR1C1, PPAR*β*/*δ* or NR1C2, and PPAR*γ* or NR1C3) share a high degree of structural similarity with all members of the nuclear hormone receptor superfamily [4–6]. The cellular and systemic roles that have been attributed to PPARs extend far beyond the control of hepatic peroxisome proliferation in the rodents after which they were initially named [2, 3, 7]. PPARs exhibit isotype-specific tissue expression patterns, with PPAR*α* expressed at high levels in organs with a significant catabolism of fatty acids, PPAR*β*/*δ* in all cell types analyzed so far with levels depending on the extent of cell proliferation and differentiation, and with PPAR*γ* found at high levels in the adipose tissues and lower levels in colon, immune cells, and other tissues [8]. Transcriptional regulation by PPARs requires heterodimerization with the retinoid X receptor (RXR), and interactions with coregulator complexes [9–11]. When activated by a ligand, the PPAR:RXR dimer controls transcription via binding to the peroxisome proliferator response element (PPRE) in the regulatory region of target genes [9]. The selective action of PPARs in different tissues results from the combination, at a given time point, between expression levels of each of the three PPAR and RXR isotypes, affinity for a specific regulatory PPRE, ligand production by lipid-modifying enzymes, and cofactor availabilities [12].

## 2. PRODUCTION OF ENDOGENOUS PPAR LIGANDS

**Figure 1:**
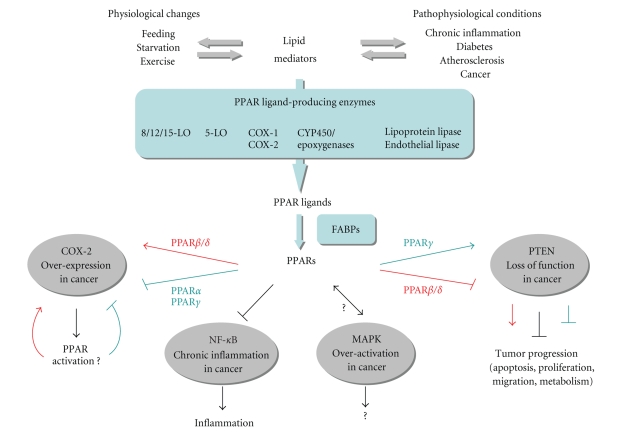
PPARs are mediators of lipid signaling in inflammation and cancer. Lipid mediators originate from and participate in the control of physiological and pathophysiological situations. Many lipid-modifying enzymes are involved in the production of PPAR ligands. The cyclooxygenases (COX), lipoxygenases (LO), epoxygenases/cytochrome (CYP)/P450s enzymes, and the lipases use either fatty acids, triglycerides, or phospholipids as substrates to generate PPAR ligands, which are guided to their receptors by the cytoplasmic fatty acid binding proteins (FABPs). PPARs translate these lipid signals into responses, which maintain energy homeostasis, regulate inflammation and modify tumor growth. Among the pathways involved in inflammation and cancer, PPARs interact with COX2, NF-*κ*B, MAPKs, and PTEN. PPAR*α* and *γ* inhibit COX2 expression, thereby reducing the production of their own ligands. Conversely, PPAR*β*/*δ* is thought to activate COX2 expression, generating a positive feedback loop by increasing the production of PPAR ligands. PPARs reduce inflammation by inhibiting NF-*κ*B, a major pathway that links chronic inflammation to cancer promotion. Several modes of interactions between PPARs and MAPKs have been reported, but the relevance and consequences of such crosstalks are unclear. Finally, PPAR*β*/*δ* and *γ* decrease and increase the expression of the tumor suppressor PTEN (phosphatase and tensin homologue deleted from chromosome 10), respectively. PPAR*γ* activation of PTEN is thought to potentiate its tumor suppressor function, whereas PPAR*β*/*δ* would have the opposite effect.

The prevalent point of view today is that the three PPAR isotypes function, in a broad sense, as lipid sensors that translate lipid signals from different origins into responses whose aim is to maintain energy homeostasis, in response to the different physiologic challenges to which the body is exposed. However, the connection between lipid metabolism pathways and PPAR responses was only recently unveiled. The production and nature of the endogenous ligands or mediators of PPAR activation have not been well characterized although it is known that many lipid-modifying enzymes are involved. The pathways that generate these lipid signals from fatty acids, which also serve as PPAR ligands, are recapitulated in Figure 1.

*ω*-3 and *ω*-6 polyunsaturated fatty acids are stored in membrane phospholipids and lipid bodies, and are released by cytosolic phospholipase A2 (cPLA2) [13]. *ω*-6 fatty acids, predominantly arachidonic acids, are abundant in the western diet and they are often converted to leukotrienes, prostaglandins, and other cyclooxygenase or lipoxygenase products [13]. They regulate cellular functions with inflammatory, atherogenic, and prothrombotic effects [13]. The *ω*-3 fatty acids, such as docosahexaenoic acid and eicosapentaenoic acid, are also substrates for cyclooxygenases and lipoxygenases. Interestingly, *ω*-3 fatty acid-derived eicosanoids antagonize the proinflammatory effects of *ω*-6 fatty acids by downregulating inflammatory and lipid synthesis genes, and by stimulating fatty acid degradation [13]. Many eicosanoids bind to PPARs and control tissue homeostasis and inflammation [3, 14].

The epoxygenases are a group of microsomal cytochrome P450s (CYP) enzymes that convert arachidonic acid to epoxyeicosatrienoic acids (EETs), which function primarily as autocrine and paracrine mediators in the cardiovascular and renal systems [15]. These mediators, which are unstable and are rapidly metabolized in most tissues, have important roles in cellular migration and proliferation, and in inflammation. Although their mechanism(s) of action is not fully understood, the epoxygenase pathway can generate potent ligands for the PPARs, which participate in antiatherogenic, antithrombotic, and cardioprotective processes that may be targeted by new therapeutic developments in vascular and inflammatory disorders [16].

The various lipases have unique pattern of expression, distinct biological actions, and preferred substrate from which they release diverse products [17]. They preferentially hydrolyze triglycerides versus phospholipids, and use lipoproteins, such as very low-density lipoproteins (VLDLs), low-density lipoproteins (LDLs), and high-density lipoproteins (HDLs), as substrates [17]. Hydrolysis of triglycerides within triglyceride-rich lipoproteins by the lipoprotein lipase (LPL) results in the transfer of lipids and apolipoproteins to HDLs. In turn, hepatic lipase (HL) hydrolyzes HDL triglyceride and phospholipids, generating smaller lipid-depleted HDL particles. Finally, endothelial lipase (EL) might hydrolyze HDL phospholipids, thus promoting HDL catabolism [17]. Lipases generate various lipolytic products such as fatty acids with different chain lengths and degrees of saturation, as well as other molecules such as monoacylglycerol. While fatty acids can be oxidized in order to gain energy, or alternatively stored in fat, they can also direct transcriptional responses. PPAR activation, as a consequence of lipolysis, underscores a key role of the functional interplay between lipases and lipoproteins. It was reported that LPL acts on circulating lipoproteins to generate PPAR*α* ligands that induce endothelial vascular cell adhesion molecule 1 (VCAM1) [18]. LPL can release HODEs, which are known as PPAR*α* agonists, from electronegative LDL, thereby reversing the proinflammatory responses of this lipoprotein. Similarly, HDL hydrolysis, and to a lesser extent hydrolysis of LDL and VLDL, by EL can also activate PPAR*α* [19, 20]. In macrophages, VLDL regulates gene expression through activation of PPAR*β*/*δ*, an activation that depends on the release of the VLDL triglycerides by LPL [21]. An additional lipase, named as adipose triglyceride lipase, desnutrin, iPLA2*ζ*, or transport secretion protein 2, was identified more recently. It increases the availability of fatty acids from VLDL, resulting in increased PPAR*β*/*δ* activity [22–24]. Obviously, the combination of a variety of lipases and lipoproteins and the resulting distribution in the organism of fatty acids and their often short-lived derivatives did not enable a precise characterization of their impact on PPAR functions as a whole. Furthermore, activation of PPARs by ligands produced by the different lipid signaling enzymes can lead to a feedback stimulation or inhibition of the expression of these enzymes (see Section 3).

## 3. GUIDING LIGANDS TO PPARs: ROLES OF FABPs

Both fatty acid binding proteins (FABPs) and retinoic acid binding proteins (CRABPs) belong to an evolutionarily conserved family of intracellular proteins [25]. Various functions have been attributed to these proteins, including cellular uptake and transport of fatty acids, the targeting of fatty acids to specific metabolic pathways, and the regulation of gene expression and cell growth [26]. Interestingly, FABPs are thought to deliver ligands to the PPARs. For instance, specific interactions with fatty acid-loaded adipocyte FABP (FABP4) and keratinocyte FABP (FABP5) selectively enhance the activity of PPAR*γ* and PPAR*β*/*δ*, respectively [27]. In this function, FABPs relocate to the nucleus when bound to ligands that are selective for the PPAR isotype they activate, and thus FABPs mediate the transcriptional activities of their own ligands. Retinoic acid receptors (RARs) belong to the same type-2 class of receptors as PPARs in the nuclear receptor superfamily [12]. A coevolution between the fatty acid and retinoid-binding protein families and the RAR and PPAR families can be postulated, which has promoted the emergence of a mechanism for directing a ligand to the appropriate receptor. The two associated systems, FABPs-PPARs and CRABPs-RAR, show some promiscuity at the expense of specificity, but in favor of an increased diversity in transcriptional responses. Depending on the ratio of FABP5 to CRABP-II, RA activates RAR or PPAR*β*/*δ*. Surprisingly, when the FABP5-to-CRABP-II ratio is high, RA serves as a physiological ligand for PPAR*β*/*δ*, which broadens the spectrum of physiological regulation due to the activity of this receptor in an unexpected way [28, 29]. The key issue raised by these studies concerns the importance of the role of directed ligand transport in nuclear receptor activation, and ligand-dependent crosstalk between different receptor types [29]. Overruling ligand selectivity between receptor categories by this mechanism might promote a promiscuity that may contribute significantly to the pleiotropic effects of key members of the nuclear receptor superfamily [28, 29].

Similarly to the genes encoding lipid-signaling enzymes, the expression of FABPs is controlled by PPARs in specific situations. L-FABP is highly expressed in the liver and small intestine, where it plays an essential role in controlling cellular fatty acid flux. Its expression is increased by both the fibrate hypolipidemic drugs and LCFAs. The different PPAR isotypes (*α*, *β*/*δ*, and *γ*) promote the upregulation by FAs of the gene encoding L-FABP in vitro, while PPAR*α* is an important regulator of L-FABP in the liver, but not in the intestine [30, 31]. In contrast, only PPAR*β*/*δ* is able to upregulate the gene encoding L-FABP in the intestine of PPAR*α*-null mice. Thus, PPAR*β*/*δ* contributes to metabolic adaptation of the small intestine to changes in the lipid content of the diet [30, 31]. In summary, FABPs bind PPAR ligands within the cytoplasm, channel this cargo to the respective nuclear receptors, and by so doing influence their activation, which sometimes regulates their own expression [32].

## 4. PPARs IN INFLAMMATION AND CANCER

Although acute inflammation is a necessary process aimed at protecting the organism after an injury or an infection, unresolved chronic inflammation may promote cancer formation by providing an appropriate environment for tumor growth [33, 34]. Mechanisms that link inflammation and cancer have only recently been studied, but epidemiological studies show a convincing association between them (see [33–36] and references therein). For example, hepatitis is often followed by the development of hepatocarcinoma, ulcerative colitis is a risk factor for colon cancer, and inflammation due to infection by *Helicobacter pylori* precedes the majority of gastric cancers [34]. In the lungs also, the risk of developing lung cancer is higher in patients suffering from asthma or from chronic bronchitis [37, 38].

The role of immune cells in tumor development is not yet fully understood. Although inflammatory mediators may promote cancer development, immune cells can also secrete cytokines that can limit tumor progression [33–35]. Data collected from mouse models suggest that the role of the immune system in cancer is likely to depend on the profile of cytokines secreted by the immune cells. Modifying this profile may contribute to the development of new treatments [33]. Based on present knowledge, the NF-*κ*B and COX2 pathways have emerged as important links between inflammation and cancer (reviewed in [36, 39–42]). Consistent with inflammation and COX2 favoring the development of tumors, long-term use of NSAIDs, albeit at relatively high doses, prevents colorectal tumor development [43].

The roles of PPARs in tumor development are still unclear and their pro- or anticarcinogenic effects remain open to discussion (reviewed in [7, 44]). PPAR activity has been associated with numerous cancer types in organs such as the liver, colon, skin, prostate, breast, and lung (reviewed in [7, 45]). The mechanisms reported so far suggest that the anticarcinogenic activity of PPARs is due to direct effects in the cancer cells themselves, such as inhibition of the cell cycle, activation of cell differentiation, or cell death (reviewed in [7, 45]). But in addition to these functions, one can speculate that PPARs may have non-cell autonomous effects by acting on the tumor environment. In fact, PPARs regulate inflammatory processes [3, 46, 47], and they fulfill vital regulatory functions in cells that are important components of the tumor stroma, such as immune or endothelial cells [35, 48–51]. In line with the link between inflammation and cancer promotion, we provide below an overview of PPARs' involvement in organs in which inflammatory pathways and cancer development are known to have been connected, namely, the skin and the digestive tract.

### 4.1. Skin, inflammation, and cancer

An analysis of various models of PPAR activation or invalidation shows that PPARs are not absolutely indispensable for normal epidermal maturation and renewal, but that they accelerate mouse and human keratinocyte differentiation, as well as mouse epidermal barrier recovery after disruption (reviewed in [52, 53]). In addition, PPAR*α* and PPAR*β*/*δ* activation regulates human hair follicle survival and mouse hair follicle growth, respectively, whereas the roles of PPARs in the sebaceous glands remain unclear (reviewed in [52]). 

After an injury, skin repair involves the recruitment of inflammatory cells, the migration and proliferation of keratinocytes, activation of dermal fibroblasts, and angiogenesis [54]. Though undetectable in the interfollicular epidermis of healthy rodent skin, the expression of PPAR*α* and PPAR*β*/*δ* is reactivated in the epidermis at the edges of skin wounds [55]. The expression of PPAR*α* is upregulated early after the injury, but the signal involved is unknown. The study of genetically modified mice showed that no, or low, PPAR*α* activity results in impaired inflammatory reaction, which causes a transient delay in healing [55, 56]. The upregulation of PPAR*β*/*δ* expression, as well as the production of an unknown endogenous agonist, is triggered by proinflammatory cytokines, such as TNF-*α* [57], whereas TGF*β*-1 signaling is responsible for the repression of inflammatory-induced PPAR*β*/*δ* expression at the end of the healing process [58]. The completion of skin healing in the PPAR*β*/*δ*-null animals is delayed, mostly because of impaired epithelialization due to apoptosis and defects in keratinocyte adhesion and migration [55, 59, 60]. Consistent with decreased healing efficiency in its absence, prolonged expression of PPAR*β*/*δ* accelerated wound closure [61, 62], whereas premature downregulation of PPAR*β*/*δ* expression temporarily delayed wound closure [62]. In summary, PPAR*α* and PPAR*β*/*δ* both promote the healing of skin wounds. PPAR*α* prevents exacerbated early inflammation, while PPAR*β*/*δ*, whose expression and activity are increased by inflammatory cytokines, enhances keratinocyte survival and migration.

 Inflammatory skin disorders are usually characterized by keratinocyte hyperproliferation and aberrant differentiation, as observed in psoriasis [63, 64]. Moreover, numerous lipid molecules, which are potent activators of PPARs, are produced in the psoriatic lesions where they accumulate [65]. Consistent with stimulated expression by inflammatory cytokines after skin injury in the mouse [57], the PPAR*β*/*δ* levels are particularly high in the hyperproliferative lesional skin of psoriatic patients [66], while those of PPAR*α* and PPAR*γ* remain unchanged, or even decrease [65, 66]. Overall, PPAR activation reduces inflammation in skin disorders [53]. It is well documented that PPAR*α* activation is beneficial in mouse models of hyperproliferative epidermis [67], in models of irritant and allergic dermatitis [68], and in a model of atopic dermatitis [69]. Interestingly, PPAR*α* may be the molecular target of the antiallergic and anti-inflammatory effects of palmitoylethanolamide, a natural fatty acid derivative present in murine skin [70]. PPAR*γ* activation also has beneficial consequences in various models of psoriatic skin, such as in organ cultures, in a model of human psoriatic skin transplant, and in murine models of keratinocyte hyperproliferation [71, 72]. Despite these promising studies in models of psoriatic skin, PPAR*α*, PPAR*β*/*δ*, or PPAR*γ* activation did not improve skin homeostasis when locally applied on psoriatic plaques [73, 74]. However, PPAR*γ* agonists thiazolidinediones efficiently normalized skin homeostasis when orally administrated to patients suffering from psoriasis (reviewed in [75, 76]), suggesting that their beneficial effects are most likely due to systemic anti-inflammatory functions of PPAR*γ*. 

The skin is constantly exposed to many types of aggression, including carcinogens such as xenobiotics or UV. Much remains to be explored regarding PPAR functions in skin cancers, either squamous or basal cell carcinomas (tumors of keratinocyte origin) or melanomas (tumors of melanocyte origin) (reviewed in [52]). Activation of PPAR*α* and PPAR*γ* reduces proliferation and stimulates differentiation of cultured melanocytes [77, 78]. Several PPAR*γ* agonists inhibit the proliferation of human malignant melanomas [79], and the PPAR*α* agonist fenofibrate has antimetastatic effects on melanoma tumors in vivo in a hamster model [80]. Interestingly, combined treatment with the PPAR*γ* agonist pioglitazone, the COX2 inhibitor rofecoxib, and angiostatic chemotherapy stabilized or even reversed chemorefractory melanoma progression, though in only 11% of the treated patients [81]. In a search for genetic factors that may increase melanoma risk, correlation between PPAR*γ* variants and melanoma development in a Caucasian population indicated that PPAR*γ* polymorphisms are an unlikely risk factor for melanoma development in this population [82]. In tumors of keratinocyte origin, increased expression of PPAR*β*/*δ* was reported in head and neck squamous carcinoma [83]. In a mouse model of DMBA/TPA-induced skin tumors, PPAR*β*/*δ*-null animals showed enhanced tumor formation, suggesting that PPAR*β*/*δ* attenuates tumor development. A possible mechanism of this effect is that, by activating the expression of ubiquitin C, PPAR*β*/*δ* activates the ubiquitin degradation pathway that is critical for the breakdown of many proteins involved in cell cycle progression [84]. Another proposed mechanism is the downregulation by PPAR*β*/*δ* of protein kinase C*α* (PKC*α*) activity, thereby also inhibiting keratinocyte proliferation [85]. However, the selective ablation of PPAR*β*/*δ* in keratinocytes did not have any incidence on the development of DMBA/TPA-induced skin tumors, suggesting that PPAR*β*/*δ* may exert its tumor modifier activity by acting on the tumor environment [49, 86]. It is worth noting that PPAR*α* activators prevented DMBA/TPA-induced skin tumors when locally applied to mouse skin [87], and reduced UV-induced inflammation in human skin, which is a risk factor for further development of UV-induced skin cancers [88]. On the contrary, the activation of PPAR*γ* did not prevent the development of UV- or DMBA/TPA-induced skin tumors [89], despite increased susceptibility of PPAR*γ*+/− and keratinocyte-selective PPAR*γ*-null mice to DMBA-mediated carcinogenesis [86, 90]. Finally, UV treatment of a human keratinocyte cell line induced the production of an unknown PPAR*γ* activator [91], but the relevance of this observation remains unclear.

 Taken together, these many observations underscore the implications of PPARs in inflammatory skin disorders, UV-induced inflammation, and tumor development. So far, PPAR*γ* activation in patients has proven efficient to treat psoriasis, but other therapeutical applications remain to be explored and defined, particularly in the field of carcinogenesis. 

### 4.2. Digestive tract inflammation

Inflammatory bowel diseases (IBDs) are inflammatory diseases affecting the small or the large intestine [92]. Crohn's disease and ulcerative colitis are the best known forms of IBDs although their causes remain unclear. In their acute phase, IBDs are characterized by acute inflammation, involving the recruitment of immune cells and an elevated production of cytokines. Under chronic conditions, abnormal intestinal epithelium morphology and scarring develop. In various animal models of IBD, the activation of PPAR*α* or PPAR*γ* has anti-inflammatory effects in the intestine, resulting in decreased production of inflammatory markers and slower progression of colitis [93–96]. In these models, PPAR*γ* is the best studied isotype. With the exception of one contradictory study showing that long-term pretreatment with a PPAR*γ* agonist aggravated colitis [97], the preventive activation of PPAR*γ* was efficient, whereas the efficacy of ligand administration after the onset of the disease was dependent on the levels of PPAR*γ* [95, 98–100]. PPAR*γ* activation also prevented colon damage caused by immobilization-induced stress [101]. Conversely, enhanced susceptibility to colitis was observed in mice with reduced PPAR*γ* levels or activity [95, 102–105]. The bases of the protective action of PPAR*γ* in colitis are reduced proinflammatory cytokine production, attenuated expression of ICAM-1 and COX-2, inhibition of NF-*κ*B and JNK/p38 MAPK, and modification of immune cell activity [44, 95, 98, 99, 102, 105–107]. In patients suffering from active ulcerative colitis, a twelve-week treatment with the PPAR*γ* agonist rosiglitazone efficiently cured four out of fifteen patients [108]. Furthermore PPAR*γ* is thought to be one of the molecular targets underlying the beneficial anti-inflammatory effect of 5-aminosalicylic acid, a drug widely used to treat inflammatory bowel diseases (IBDs) [109]. Together, these treatments confirm PPAR*γ* as a potential target in IBDs. The beneficial role of PPAR*γ* activation in inflammatory diseases of the digestive tract may not be limited to the intestine, but seems to extend to gastritis and pancreatitis, an inflammation of the gastric mucosa and pancreas, respectively. In several models of gastritis or gastric ulcers, activation of PPAR*γ* attenuates mucosa damage and accelerates healing, via reduction of inflammation, apoptosis, and lipid peroxidation [110–115]. As in the stomach, PPAR*γ* activity is beneficial in various animal models of pancreatitis, reducing inflammation, restoring exocrine pancreas functions, and limiting chronic pancreatitis development [116–121].

In addition to its already mentioned anti-inflammatory effects, PPAR*α* protects the intestine from colitis-induced permeability [122]. So far, the benefits of PPAR*β*/*δ* activation in colitis are poorly documented [44]. One report suggested that PPAR*β*/*δ*-null mice exhibit more severe damage in a model of DSS-induced colitis, whereas a PPAR*β*/*δ* agonist had no protective or deleterious effect when administrated to PPAR*β*/*δ*-wt or -null animals [123]. This observation suggests not only that PPAR*β*/*δ* protects wt animals against DSS-colitis, but also that this protective effect may be ligand-independent or triggered by a so far nonidentified ligand.

The liver is an additional target organ of PPARs for the control of inflammation. Prolonged liver inflammation, which is deleterious, usually activates hepatic stellate cells (HSCs), also known as Ito cells or lipocytes, which proliferate, transdifferentiate into myofibroblasts, and produce excess extracellular matrix, finally leading to severe fibrosis and end-stage cirrhosis [124]. Animal models suggest that limiting, or even reversing, fibrosis may be possible by reducing inflammation, enhancing HSC apoptosis, blocking HSC transdifferentiation, or stimulating ECM degradation [124]. Although PPAR*β*/*δ* activation seems to enhance fibrosis via activation of HSC [125], increasing PPAR*α* or PPAR*γ* activity appears to have antifibrotic effects. PPAR*α* reduces inflammation and oxidative stress [126, 127], and PPAR*γ* decreases HSC proliferation, reverses their profibrotic activity, and counteracts the TGF*β*1-induced production of collagen [128–136]. Recently, PPAR*γ* activity in human hepatic stellate cells has been shown to be inhibited by acetaldehyde, the major product of ethanol oxidation and one of the main mediators of alcohol-induced liver fibrosis [137].

In conclusion, manipulating the balance of PPAR isotype activities is an interesting therapeutic concept when used to control inflammation of the digestive tract and associated glands.

### 4.3. Digestive tract and cancer

As the literature includes extensive recent reviews on the interaction between PPARs and Wnt/Apc, known to play a major role in colorectal cancer progression [7, 138], this paragraph will focus on data dealing with chronic inflammation as a risk factor for colon carcinogenesis. Inflammatory bowel diseases, particularly ulcerative colitis, increase the risk of colorectal cancer in patients [139]. As discussed above, PPAR*γ* activation has protective effects in animal models of ulcerative colitis (reviewed in [140]). Moreover, activation of PPAR*α* and PPAR*γ* in rodents reduced the formation of aberrant crypt foci, a risk factor for colon cancer [94]. However, the PPAR*γ* agonists pioglitazone and rosiglitazone had no effect on the development of tumors in a mouse model of azoxymethane/dextran sodium sulfate-induced colon cancer, whereas in the same study the anti-inflammatory 5-ASA reduced the number and the size of the tumors [141], showing that PPAR*γ* is certainly not the only target of 5-ASA. However, in a different study, COX2 inhibitors, the PPAR*γ* agonist troglitazone and, to a lesser extent, the PPAR*α* agonist bezafibrate, reduced the development of adenocarcinoma in a mouse model of azoxymethane/dextran sodium sulfate-induced colon cancer [142, 143].

Chronic inflammation finally leading to cancer may also arise from infections, as in the stomach where infection by *Helicobacter pylori* is a common risk factor for gastric cancer [144]. PPAR*γ* expression is increased in gastric epithelia infected by *Helicobacter pylori*. The consequences of upregulated PPAR*γ* expression are unknown, but it may contribute to reducing inflammation [145]. The treatment of gastric cancer patients with the COX2 inhibitor rofecoxib correlated with increased levels of PPAR*γ* in the tumor [146]. An epidemiological study performed in a restricted region of Japan suggested that the Pro12Ala variant of PPAR*γ*, which is less active than the wt protein, might be associated with increased risk of gastric cancer [147].

Pancreatic cancer is still lethal in most cases, due to the lack of early markers and specific symptoms and because of aggressive tumor growth and resistance to treatments [148]. While PPAR*γ* activation shows beneficial anti-inflammatory effects in the pancreas, the consequences of such activation in patients with pancreatic cancer are unknown. In vitro data show that PPAR*γ* inhibits pancreatic cell proliferation, which would be beneficial, but also suggest that PPAR*γ* may activate angiogenesis through induced VEGF expression, which would be detrimental (reviewed in [148]). In one in vivo study, however, the PPAR*γ* agonist pioglitazone prevented cancer in a hamster model [149]. In human patients, a high level of PPAR*γ* expression correlated with high-grade pancreatic carcinoma [150]. The mechanism responsible for this effect remains unknown.

### 4.4. Age-related diseases

Oxidative stress and inflammation increase with age, and further enhancement by environmental factors is thought to favor the development of age-related diseases and cancers. Although this is not fully clear in human, slight caloric restriction diet may retard these processes. The roles of PPARs in age-related inflammation and associated diseases have been reviewed recently in [151–153]. In short, PPARs are thought to be involved in age-related inflammation, caloric restriction physiology, and longevity. Increased inflammation levels during aging are correlated to decreased PPAR activity. Conversely, administration of the PPAR*α* activator Wy14,643 improved the redox balance and reduced inflammation in aged mice [154, 155]. A similar inhibition of age-related inflammation was observed in rat kidney after feeding with a PPAR*γ* agonist [156]. Interestingly, among flavonoids found in fruits and vegetables, which have been associated with decreased risk of inflammation-mediated diseases, some are PPAR*γ* agonists that are known to decrease proinflammatory mediator production. For instance, curcumin, a naturally occurring compound in turmeric, has been used in India for centuries as an anti-inflammatory agent. It is thought to be a PPAR*γ* activator and was suggested to have beneficial effect on colorectal cancer when taken on a daily basis [152, 157].

## 5. CROSSTALK BETWEEN PPARs AND PATHWAYS RELEVANT TO CANCER AND INFLAMMATION

It is obvious from the above that PPARs interact with numerous pathways involved in cancer development (reviewed in [7, 45, 158]). For instance, PPAR*α* regulates the expression of miRNA let-7C in hepatocytes, a tumor suppressor gene that regulates cancer cell proliferation. PPAR*β*/*δ* is a downstream target of two pathways often involved in colon cancer development, namely, the Ras and the APC-*β*-catenin pathways. PPAR*β*/*δ* also controls the PTEN/Pi3K/Akt pathway, whose actors are often associated with cancer, and promotes cell migration via activation of the Rho-GTPases [60]. Finally, PPAR*γ* activation can induce growth arrest, differentiation, or apoptosis in many cancer cells [7].

In the next sections, we summarize the interaction of PPARs with the main pathways involved in the control of inflammatory responses and cancer development [3, 46].

### 5.1. COX2 as a link to lipid mediators

Cyclooxygenases (COX) are the enzymes that catalyze the first steps of the production of prostaglandins from arachidonic acid. The COX1 isoform is constitutively expressed in most tissues, whereas the expression of COX2 is induced in inflamed tissues and in tumors. Genetic, epidemiological, and pharmacological evidence supports the hypothesis that elevated COX2 activity is involved in tumor progression (reviewed in [159–161]). Laboratory experiments as well as clinical studies have shown that COX2 inhibitors are promising antitumoral compounds to combine with other anticancer treatments. However, there is a need to develop new compounds with reduced risk of cardiovascular side effects (reviewed in [40, 159, 161, 162]). Antitumoral activity of COX2 inhibitors most probably results from a combination of effects on angiogenesis, apoptosis, tumor cell invasiveness, and inflammation. Interestingly, PPAR*α* and *γ* activation may help in inhibiting the activity of COX2 by reducing its expression. PPAR*α* agonists prevented PMA-induced expression of COX2 and VEGF [163], and the PPAR*γ* agonist ciglitazone decreased the expression of COX2 and cJun in a colorectal cancer cell line [164]. COX2 can also modify PPAR activity since some of the COX-2-produced fatty acid derivatives are PPAR activators. COX2 has been proposed to modify the activity of PPAR*β*/*δ* in colorectal cancer by producing activators such as PGI2 [165–167] or PGE2, which indirectly increase PPAR*β*/*δ* activity [168]. In human cholangiocarcinoma cell lines, activation of PPAR*β*/*δ* was shown to increase cell proliferation by increasing the expression of COX2 and thus the production of PGE2 [169]. In this model, PGE2 is meant to subsequently activate PPAR*β*/*δ* indirectly via cPLA2*α*, thereby triggering a positive feedback loop controlling cholangiocarcinoma cell proliferation. Inhibiting COX2 is likely to result in decreased PPAR activity. This was in fact demonstrated in hair follicle growth of murine skin, during which inhibition of COX2 replicates the phenotype of PPAR*β*/*δ*-null animals [170]. However, increased PPAR*γ* activity by COX2 inhibitors was also reported, although the mechanism remains unknown (reviewed in [148]). The COX2 and PPAR pathways are certainly interconnected, but to what extent the PPAR activity contributes to the COX2 cancer promotion function is unclear. However, drug-combined modification of PPAR activity in inflammation and cancer is an interesting therapeutic prospect.

### 5.2. NF-*κ*B links inflammation to cancer

The NF-*κ*B pathway is an important link between inflammation and cancer (see [41]; reviewed in [36, 42]). The three PPARs are able to antagonize this pathway, via their transactivation or transrepression activities, thereby leading to the repression of several genes involved in inflammation [3, 44, 47]. In colon cancer cell lines, the PPAR*γ* agonist 15d-PGJ_2_ attenuated the production of IL-1*β*-induced IL-8 and MCP-1 by inhibition of NF-*κ*B activity [96], and induced apoptosis via NF-*κ*B and Bcl-2 [171]. In the liver, the disruption of NF-*κ*B signaling resulted in the suppression of PPAR*α*-increased expression during a high-fat diet, whereas, in parallel, an increase in PPAR*γ* expression was observed. In these mice, liver steatosis (a consequence of decreased FA oxidation and increased expression of genes involved in lipogenesis), inflammation, and development of liver cancer were aggravated [172]. Animal and preclinical studies showed that an *ω*-3 fatty acid supplement to the diet should provide a useful complement to cancer therapy, slowing down progression of various tumors and improving patients' quality of life [173]. Among the mechanisms proposed for these beneficial effects, *ω*-3 fatty acids repress the NF-*κ*B function and Bcl-2 expression, which in turn leads to decreased COX2 expression and restoration of functional apoptosis [173]. In addition to PPARs regulating the activity of NF-*κ*B, the p65 subunit of the latter was shown to inhibit the transcriptional activity of PPAR*γ* on adipocyte gene expression [174] and of the three PPARs in transfected keratinocytes [65], suggesting that a reciprocal regulation between the two pathways exists.

### 5.3. MAPK pathway as a major player in carcinogenesis

The MAPK pathway is activated by cytokines, and its overactivation is found in the vast majority of cancer cells and tumors (reviewed in [175]). Phosphorylation of PPAR*α* and PPAR*γ* by this pathway increases or decreases their transcriptional activity, respectively (reviewed in [9, 176]). The physiological impact of the regulation of PPAR activity through phosphorylation has mostly been addressed for PPAR*α* and *γ* regarding insulin signaling and fatty acid metabolism, but the impact of this modification on inflammation or cancer is currently not documented [9, 176]. Nevertheless, PPAR and MAPK crosstalk has been described in immune or cancer cells. In its unliganded form, PPAR*α* suppressed p38 MAPK phosphorylation in CD4(+) T cells. Ligand activation reversed this inhibition, resulting in the expression of the transcription factor of T cells (T-bet), a marker of Th1 inflammatory responses [177]. The PPAR*γ* agonist rosiglitazone attenuated TNBS-induced colitis via inhibition of the activity of the MAPKs p38 and the c-Jun N-terminal kinase (JNK), and of NF-*κ*B, thereby limiting the expression of proinflammatory genes [95]. In a human colon cancer cell line, PPAR*γ* activation was reported to increase the expression of caveolin1, a protein that is linked to cancer development [178]. This induction seemed to result from an activation of the MAPK pathway by PPAR*γ*. In another study, the activation of PPAR*γ* in turn activated the Rho-GTPase/MEK1/ERK1/2 cascade, resulting in morphological changes and increased motility in rat intestinal epithelial cells [179]. In lung cancer cell lines, the PPAR*γ* agonist troglitazone induced cell differentiation, probably via activation of Erk1/2 [180, 181]. In addition, the Erk5-dependent activation of PPAR*γ* seemed to be responsible for the antitumorigenic effect of the Wnt signaling pathway [182]. PPAR*β*/*δ* also interacts with the MAPK pathway. When activated by TNF*α*, the MAPK pathway induced the expression of the PPAR*β*/*δ* gene in inflamed keratinocytes [57]. Once activated by a ligand produced in parallel, PPAR*β*/*δ* facilitates keratinocyte survival and migration. Interestingly, both the expression of PPAR*β*/*δ* and the activity of the MAPK pathway are elevated in many tumors [7, 175]. Whether the expression of PPAR*β*/*δ* is stimulated by this pathway in cancers remains to be investigated. Finally, anti-inflammatory effects of the MEK5/Erk5 pathway in a muscle cell line are due to inhibition of NF-*κ*B and are thought to involve PPAR*β*/*δ* activation [183].

Crosstalk between PPARs and MEKs, the upstream regulators of the MAPK, has also been described [184]. It has been suggested that MEK1 interacts with PPAR*γ*, thereby causing PPAR*γ* delocalization from the nucleus to the cytoplasm [185]. Interestingly, PPAR*γ* was described as mainly cytoplasmic in human biopsies of salivary duct carcinoma and breast cancer [186, 187]. Although the significance of this shuttling is unclear, it should decrease PPAR*γ* transactivation functions.

### 5.4. PTEN/Pi3K pathway and its target mTOR

The phosphatase and tensin homologue deleted from chromosome 10 (PTEN) is a tumor suppressor whose activity is lost in many human cancers. PTEN is a lipid and protein phosphatase whose main substrate is the PIP3 produced by the Pi3K. Through its phosphatase activity, PTEN antagonizes PiK3 activity and inhibits the Pi3K/Akt pathway involved in the regulation of apoptosis, cell proliferation and growth, and metabolism [188, 189]. The mammalian target of rapamycin (mTOR), one of the targets of the PTEN/Pi3K pathway, is a conserved kinase that regulates central cellular functions in response to environmental signals, such as transcription and translation, mRNA and protein turnover, or autophagy (reviewed in [190, 191]). Impaired mTOR pathway is often associated with tumorigenesis [1]. PPAR*β*/*δ* was shown to indirectly inhibit the expression of PTEN in keratinocytes, thereby activating the Pi3K/Akt pathway, which enabled keratinocyte survival [59]. In lung carcinoma cells, the activation of PPAR*β*/*δ* stimulated cell proliferation, via decreased expression of PTEN and activation of NF-*κ*B and Pi3K/Akt [192, 193]. While PPAR*β*/*δ* decreases PTEN expression, PPAR*γ* activation has the opposite effect. In a model of allergic inflammation in mouse lung, PPAR*γ* agonists decreased inflammation, most probably via increased PTEN expression, and reduced PiP3 levels as well as Akt and NF*κ*B activities [194]. Treatment of lung carcinoma cell lines with rosiglitazone decreased proliferation via PPAR*γ*-dependent upregulation of PTEN and inhibition of Akt activity, and also via PPAR*γ*-independent inhibition of the mTOR pathway [195, 196]. PPAR*γ*-independent inhibition of mTOR by TZD was also reported in keratinocytes [197]. In this model, TZD inhibited the mitogenic effect of IGF via indirect inhibition of mTOR, a mechanism which may be involved in TZD-mediated inhibition of skin tumor development in transgenic mice overexpressing IGF.

In a hepatocarcinoma cell line, PPAR*γ* activation by rosiglitazone inhibited cell migration through increased expression of PTEN [198]. Rosiglitazone also had important anticarcinogenic effects in some highly aggressive anaplastic thyroid cancer cell lines. In these cells, rosiglitazone induced apoptosis, cell cycle inhibition, differentiation, and decreased anchorage-independent growth and migration. This was at least partially due to upregulation of PTEN and inhibition of Akt activity, which antagonized IGF-1 effects necessary for the progression of thyroid cancers [199].

In summary, PPAR*β*/*δ* and *γ* are both regulators of the expression of PTEN, and interact with the mTOR pathway. PPAR*β*/*δ* decreases PTEN expression, whereas PPAR*γ* activates this tumor suppressor gene.

## 6. CONCLUSIONS

In numerous cancer types, PPARs regulate autonomous processes in tumor cells, such as apoptosis, proliferation, and differentiation, by interacting with major pathways involved in carcinogenesis. They also act on the tumor cell environment, modifying angiogenesis, inflammation, and immune cell functions (reviewed in [3, 7, 45, 48–51]). Not surprisingly, their activation has complex consequences, in which the contribution of tumor cell-autonomous versus nonautonomous mechanisms remains to be evaluated. Whether PPARs are pro- or anticarcinogenic actors is still open to discussion, and may depend not only on the origin and genetics of the tumor cell, but also on the nature of the host tissue and inflammation levels. Although the possible carcinogenic or toxic effects of PPAR activation remain an unresolved issue, PPARs nevertheless constitute valuable therapeutic targets (reviewed in [7, 200]). The use of PPAR*α* and PPAR*γ* agonists is increasing in the treatment of a constantly expanding number of diseases related to the metabolic syndrome. In this context, although their supposedly carcinogenic or toxic effects have to be carefully monitored, PPARs are important therapeutic targets. Many valuable approaches are now under investigation in order to better understand the mechanisms of adverse effects, and to develop better compounds. In vivo models, such as tissue or cell-type selective PPAR knock-out mice, as well as humanized animals carrying the human PPAR genes, will certainly help in sorting out the various actions of PPARs in inflammation and cancer. In addition, the development of selective PPAR modulators (SPPARMs), rather than PPAR full agonists, which would retain most of the benefits while reducing the adverse effects of PPAR activation, is a promising approach. For all these reasons, PPARs are certainly useful pharmaceutical targets to be explored further in the context of inflammation and/or cancer therapy.
